# Relationship between exposure to fine particulate matter and cardiovascular risk factors and the modifying effect of socioeconomic status: a cross-sectional study in Beijing, China

**DOI:** 10.3389/fpubh.2024.1398396

**Published:** 2024-07-19

**Authors:** Jing Du, Bing Shao, Yanlin Gao, Zaihua Wei, Yu Zhang, Hong Li, Jiang Li, Gang Li

**Affiliations:** ^1^Institute of Information and Statistics Center, Beijing Center for Disease Prevention and Control, Beijing, China; ^2^Beijing Key Laboratory of Diagnostic and Traceability Technologies for Food Poisoning, Beijing Center for Disease Prevention and Control, Beijing, China; ^3^Hongzheng Medical Technology Co., Ltd., Tianjin, China

**Keywords:** PM_2.5_, SES, cardiovascular risk factors, blood lipids, diabetes, HHcy

## Abstract

Accumulating research suggested that long-term exposure to fine particulate matter (PM_2.5_) is related to cardiovascular disease (CVD). However, evidence regarding the relationship between PM_2.5_ and CVD risk factors remains inconsistent. We hypothesized that this association may be partially modified by socioeconomic status (SES). To investigate the relationships and to test the modifying effect of SES, we included baseline data for 21,018 adults from September 2017 to May 2018. PM_2.5_ concentrations were determined by employing an amalgamation of linear measurements obtained from monitoring stations located near the participants' residential and workplace addresses. We assessed SES across several domains, including income, education, and occupation levels, as well as through a composite SES index. The results indicated that for every 10 μg/m3 increase in PM_2.5_ exposure, the risk of hypercholesterolemia, hyperbetalipoproteinemia, diabetes, and hyperhomocysteinemia (HHcy) increased by 7.7% [Odds ratio (OR) = 1.077, 95% Confidence Interval (CI) = 1.011, 1.146], 19.6% (OR = 1.196, 95% CI = 1.091, 1.312), 4.2% (OR = 1.042, 95% CI = 1.002, 1.084), and 17.1% (OR = 1.171, 95% CI = 1.133, 1.209), respectively. Compared to the high SES group, those with low SES are more prone to hypercholesterolemia, hyperbetalipoproteinemia, diabetes, and HHcy. Notably, the disparities in SES appear significant in the relationship between PM_2.5_ exposure and hypercholesterolemia as well as hyperbetalipoproteinemia. But for diabetes and HHcy, the modification effect of SES on PM_2.5_ shows an inconsistent pattern. In conclusion, the results confirm the association between PM_2.5_ and cardiovascular risk factors and low SES significantly amplified the adverse PM_2.5_ effect on dyslipidemia. It is crucial to emphasize a need to improve the socioeconomic inequality among adults in Beijing and contribute to the understanding of the urgency in protecting the health of vulnerable groups.

## Graphical Abstract

Association between PM_2.5_ and cardiovascular risk factors and the modifying effect of SES. 
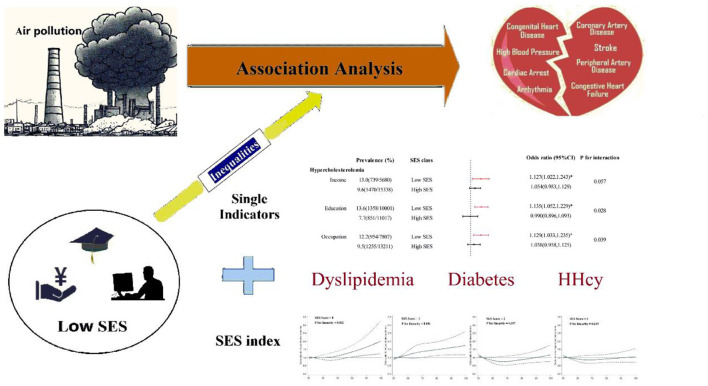

## Highlights

- PM_2.5_ exposure was significantly associated with unfavorable cardiovascular health profiles.
- In China, those with lower SES were less likely to reach their cardiovascular risk factors targets.
- Low SES significantly amplified the adverse PM_2.5_ effect on dyslipidemia.
- For diabetes and hyperhomocysteinemia, the modification effect of SES on PM_2.5_ shows an inconsistent pattern.

## 1 Introduction

Premature death and decreased quality of life in older adults are primarily attributed to cardiovascular disease (CVD) (1, 2). Cardiovascular risk factors, which predominantly include hypertension, dyslipidemia, and diabetes, play a pivotal part in the occurrence and progression of CVD (3–5). In addition, hyperhomocysteinemia (HHcy) is usually a consequence of a reduction in the activity of enzymes involved in homocysteine (Hcy) metabolism, which is generally a crucial and modifiable risk factor for CVD (6). Rapid economic growth, coupled with the increasing adoption of unhealthy lifestyles, has caused a continuous increase in the incidence of cardiovascular risk factors, which in turn has led to a substantial increase in the CVD burden in China (7).

A considerable amount of accumulating research suggested that long-term exposure to fine particulate matter (PM_2.5_) is connected to an elevated likelihood of developing CVD (8, 9). Based on biological plausibility, it is conceivable that prolonged exposure to air pollution, through the aforementioned mechanistic pathways, might induce undesirable alterations in CVD risk factors, encompassing blood lipid, glucose, and Hcy levels (10, 11). Consequently, these modifications could serve as intermediaries connecting exposures to the overt manifestation of CVD (12, 13). However, epidemiological studies associating these exposures with CVD risk factors are lacking (14, 15), and evaluations may be influenced by remaining confounders, such as socioeconomic status (SES) and nutritional status (16, 17).

Low SES, which is commonly measured in terms of low income, limited education, or primarily manual labor occupations (18, 19), has been acknowledged as a potential contributory element to cardiovascular metabolic disorders (20). Socioeconomic inequities remain barriers to the optimal management of cardiovascular risk factors (4). In addition, SES assumedly covaries with the spatial distribution of PM_2.5_ (21, 22). Therefore, it is common for air pollution epidemiology studies to consider SES as a confounding factor (23, 24); in addition, SES is likely a crucial effect modifier, and results that do not include examination of this effect modification may cause incorrect estimation of the true burden of PM_2.5_ exposure (25–28). However, the majority of the relevant literature includes populations in China or other low- or middle-income countries and provides scarce evidence that SES may modify the relationship between PM_2.5_ concentrations and cardiovascular risk factors.

Although a boom in economic development has occurred in China in recent decades, groups with severely low SES remain (29), causing serious inequity in the utilization of medical services (30). During the COVID-19 pandemic, the impacts of inequity have been more pronounced, with the greatest impact on socially vulnerable groups (31). Thus, up-to-date studies are needed to determine the status quo in China. Our objective was to address two questions related to the impact of SES on the association between PM_2.5_ exposure and cardiovascular risk factors in this study. Our first question was related to the assessment of the relationship between long-term exposure to PM_2.5_ and cardiovascular risk factors while adjusting for SES. Here, we used multiple indicators spanning three aspects of SES, namely, income, education, and occupation, to fully capture its relevance in this particular study. Our second question focused on whether individuals with low SES are more vulnerable to the pollutant-related health effects. This issue is pivotal in protecting the health of vulnerable groups and we addressed this question by testing whether SES status is a modifying effect. To fully understand the relationship between air pollution exposure and these indicators modified by different SES levels, we further constructed a composite SES index.

## 2 Methods

### 2.1 Study population

The objective of the Beijing Population Health Cohort study was to prospectively investigate the interplay between environmental and genetic factors across a broad spectrum of diseases. The study's design specifications have been outlined in a previous publication (32, 33). From September 2017 to May 2018, we used a multistage, stratified cluster method to select 215 communities or villages and 24,990 participants across all 16 districts in Beijing (see Supplementary Figure S1). After excluding 3,972 individuals due to incomplete residential information, missing SES data, or refusal to undergo a physical examination, the current analysis comprises a total of 21,018 participants. Our study protocol was approved by the Beijing Center for Disease Prevention and Control Ethics Committee [No. 2017D (6)], and all participants provided written consent after being fully informed before taking part in the study.

### 2.2 Definition of health outcomes

Blood samples were obtained from individuals after overnight fasting and were subsequently transported on the same day. A Hitachi Autoanalyzer (Type 7600, Hitachi Ltd.) was used to conduct the biochemical analysis and laboratory tests, which included the determination of glucose, total cholesterol (TC), triglyceride (TG), high-density lipoprotein cholesterol (HDL-C) and low-density lipoprotein cholesterol (LDL-C) levels, in the laboratory of the BJCDC. Dyslipidemia was defined according to the 2016 Chinese Adult Dyslipidemia Prevention Guideline (34), and hypercholesterolemia, hypertriglyceridemia, hypoalphalipoproteinemia and hyperbetalipoproteinemia were defined as TC ≥240 mg/dl, TG ≥200 mg/dl, HDL-C ≤ 40 mg/dl, and LDL-C ≥160 mg/dl, respectively. Diabetes was defined as a fasting blood glucose (FBG) level exceeding 7 mmol/L, the use of antidiabetic medications, or a confirmed diagnosis of the condition. An elevated level of Hcy, specifically a level ≥15 μmol/L, was defined as HHcy.

### 2.3 Exposure assessment

Over a period of 730 days, PM_2.5_ data from 35 air monitoring stations were meticulously gathered hourly. This comprehensive data was subsequently utilized to evaluate the long-term impacts of PM_2.5_ exposure on cardiovascular risk factors. The entire process adhered strictly to the standardized guidelines established by the State Environmental Protection Administration of China (SEPA) in 1992. For a comprehensive understanding of and further details regarding the analytical methods, quality control procedures, quality assurance practices, and outlier management strategies employed to collect the monitoring data, please see the Supplementary Method S1.

We acquired the residential and occupational addresses of every participant, allowing us to perform geocoding for each address. For every residential and occupational address, we selected a representative monitoring station based on minimal proximity. In our investigation, the average distances between the residential and occupational addresses and their respective representative monitors were 3.52 km and 3.17 km. The exposure averaged over 730 days was calculated by means of a linear aggregation of the concentrations that were measured at both locations, with the weighting based on the duration of time spent at each location:


$$\begin{array}{l} \left(\overset{730}{\underset{i}{\sum }}\overset{work\;hours}{\underset{j}{\sum }}CON_{1ij}\;+\overset{730}{\underset{i}{\sum }}\overset{home\;hours}{\underset{j}{\sum }}CON_{2ij}\right)/\left(730\;d\times 24\;h\right) \end{array}$$

where CON_1ij_ and CON_2ij_ represent the PM_2.5_ concentrations at the residential and occupational addresses, respectively, during hour *i* on day *j*. Details about the exposure algorithm have been described elsewhere (32).

### 2.4 SES

To determine the modifying effect of SES on the relationship between long-term exposure to PM_2.5_ and cardiovascular risk factors, we evaluated three distinct domains: family income, education and occupation. According to the annual disposable income per capita of Beijing residents in 2016, we grouped income into two groups: low ( ≤ 50,000 RMB/year) and high (>50,000 RMB/year). In terms of education, referring to the standards of China's higher education and previous epidemiological findings (25, 35), we have categorized education into high school education and below, and college education and above. Occupations were classified as mainly manual occupations (i.e. homemakers, crafts and related trade workers, semi-skilled and unskilled manual workers, farmers, and people in elementary occupations) and non-manual occupations (i.e. clerical, service and sales workers, professionals and managers) (36, 37). We further combined three dimensions of SES to develop a composite SES index ranging from 0 to 4 (38). The first category, with an SES level of 0, included individuals who were at low levels of all SES measures. The second and third categories included individuals who were at low levels of any one or any two of the three SES measures. The fourth SES category included individuals who were not at a low level on any SES measures.

### 2.5 Covariates

Trained personnel administered a standardized questionnaire to collect data on age, sex, SES, marital status, smoking and drinking status, the frequency of fruit consumption (one or more servings/less than one serving per day), the frequency of vegetable consumption (two or more servings/fewer than two servings per day), physical activity level (in accordance with the International Physical Activity Questionnaire) (39), and comorbidities. Hypertension was defined as a measured unaware systolic blood pressure (SBP) ≥140 mmHg or a diastolic blood pressure (DBP) ≥90 mmHg, the self-reported use of blood pressure-lowering medication or a physician diagnosis of hypertension. On the day of data collection, height and weight were measured while the individuals were barefoot and lightly dressed, and body mass index (BMI, kg/m^2^) was computed.

### 2.6 Statistical analyses

Linear mixed regression was employed to calculate the effects (b) of PM_2.5_ exposure on cardiovascular metabolic indicators in which the community or village was considered to have a random effect. For each 10-mg/m^3^ increment, after log-transforming the data to enhance linearity, the corresponding 95% confidence intervals (95% CIs) were derived by employing the formula 100 × [exp(*b*) – 1] for various health indicators including TC, TG, HDL-C, LDL-C, FBG, and Hcy levels. Odds ratios (ORs) with 95% CIs were obtained by using logistic models with respect to 10 mg/m^3^ increments of PM_2.5_ and cardiovascular risk factors. In this analysis, participants were considered as first-level units, while communities or villages treated as second-level units. Comprehensive explanations of the linear mixed regression and the two-level binary logistic regression models have been offered in prior researches (15, 40–42), as well as in the accompanying (Supplementary Method S2). We included covariates based on the hypothetical causal pathway linking PM_2.5_ exposure and cardiovascular risk factors as well as previous results from the literature. Covariates within a certain range were added gradually to the different models.

We conducted further investigations to determine whether the associations between PM_2.5_ exposure and hypercholesterolemia, hyperbetalipoproteinemia, diabetes, and HHcy were influenced by SES (comprising income, education, and occupation levels). To achieve this, we introduced an interaction term between SES and continuous PM_2.5_ exposure. Additionally, we employed restricted cubic spline method to meticulously detect the dose–response curves between PM_2.5_ exposure and cardiovascular risk factors across various SES categories.

We conducted sensitivity analyses to confirm the robustness of the present results. We first include multiple pollutants in the original regression models. Second, we evaluated the associations by excluding participants with CVD. Third, we utilized the average concentrations of air pollution during the 1-year period prior to the baseline survey as a substitution for long-term exposure measurements. Finally, we further utilized the average PM_2.5_ exposure levels estimated by the Weather Research and Forecasting (WRF) model (32, 43), spanning the 2-year prior to the baseline survey, with a spatial resolution of 0.1 degree, to comprehensively capture the variations in individual exposure as accurately as possible.

All the statistical analyses were performed using R software 4.2.1. A *P*-value of < 0.05 indicated statistical significance for a two-tailed test.

## 3 Results

The baseline characteristics of the participants in the present study are described in Table 1. Among the 21,018 participants, the average age was 46.0 (14.3) years, and 46.9% (*n* = 9,849) were men. The participants were generally nonsmokers (70.6%), nondrinkers (70.0%), and had a high level of physical activity (45.8%). The unadjusted prevalence of hypercholesterolemia, hypertriglyceridemia, hypoalphalipoproteinemia, hyperbetalipoproteinemia, diabetes, and HHcy was 10.5%, 19.1%, 13.8%, 7.4%, 12.5% and 43.5%, respectively. A comparison between the included study population and the excluded patients is available in Supplementary Table S1. Excluded participants were more likely to be younger, unmarried, non-smokers, and non-drinkers, with low education and unskilled occupation. But there were no significant differences in terms of sex, BMI and income level. Within the SES quartiles, we identified statistically significant differences in the distributions of the selected characteristics (all *P* < 0.05; see Supplementary Table S2). Furthermore, the concentrations of the four air pollutants varied slightly across the different SES classes, with a median value of 67.3 μg/m^3^ for PM_2.5_.

**Table 1:** Baseline characteristics of the study participants.

**Characteristics**	**Total (*N* = 21,018)**
Age (years), mean (±SD)	46.0 (14.3)
Male, *n* (%)	9,849 (46.9)
Married, *n* (%)	17,920 (85.3)
BMI (kg/m^2^), mean (± SD)	25.3 (7.6)
**Smoking**, ***n*** **(%)**	
Nonsmoker	14,835 (70.6)
Former smoker	1,152 (5.5)
Current smoker	5,031 (23.9)
**Drinking**, ***n*** **(%)**	
Nondrinker	14,702 (70.0)
Nonhabitual drinker	4,333 (20.6)
Habitual drinker	1,983 (9.4)
**Physical activity**, ***n*** **(%)**	
Low	5,047 (24)
Moderate	6,352 (30.2)
High	9,619 (45.8)
High fruit intake, *n* (%)	4,341 (20.7)
High vegetable intake, *n* (%)	6,072 (28.9)
**Education**, ***n*** **(%)**	
Low	10,001 (47.6)
High	11,017 (52.4)
**Income**, ***n*** **(%)**	
Low	5,644 (26.8)
High	15,374 (73.2)
**Occupation**, ***n*** **(%)**	
Mainly manual	7,807 (37.1)
Non-manual	13,211 (62.9)
Hypertension, *n* (%)	7,895 (37.6)
Diabetes, *n* (%)	2,622 (12.5)
Hypercholesterolemia, *n* (%)	2,209 (10.5)
Hypertriglyceridemia, *n* (%)	4,011 (19.1)
Hypoalphalipoproteinemia, *n* (%)	2,908 (13.8)
Hyperbetalipoproteinemia, *n* (%)	1,561 (7.4)
HHcy, *n* (%)	9,133 (43.5)
PM_2.5_ (μg/m^3^)	67.3 (10.9)

Table 2 indicates that increased exposure to PM_2.5_ is linked to the increases in TC, LDL-C, FBG, and Hcy levels. Specifically, for an increase of 10 mg/m^3^ in PM_2.5_ exposure, a corresponding increase of 0.729% (95% CI = 0.324%, 1.134%) in the TC level, 1.973% (95% CI = 1.240%, 2.712%) in the LDL-C level, 1.026% (95% CI = 0.724%, 1.330%) in the FBG level, and 1.905% (95% CI = 0.800%, 3.022%) in the Hcy level after fully adjusting for confounding factors was observed.

**Table 2:** Percent change (%, mean and 95% CI) in cardiovascular metabolic indicators associated with a 10 μg/m^3^ increase in PM_2.5_ exposure.

**PM_2.5_, μg/m^3^**	**Model 1^a^**	**Model 2^b^**	**Model 3^c^**
TC, mg/dl	0.732 (0.325, 1.139)^*^	0.738 (0.333, 1.144)^*^	0.729 (0.324, 1.134)^*^
TG, mg/dl	0.293 (−1.015, 1.617)	0.283 (−0.985, 1.566)	0.116 (−1.114, 1.362)
HDL-C, mg/dl	0.729 (−0.170, 1.637)	0.67 (−0.220, 1.568)	0.661 (−0.230, 1.556)
LDL-C, mg/dl	1.959 (1.225, 2.698)^*^	1.954 (1.220, 2.692)^*^	1.973 (1.240, 2.712)^*^
FBG, mmol/L	1.140 (0.835, 1.446)^*^	1.142 (0.837, 1.448)^*^	1.026 (0.724, 1.330)^*^
Hcy, μmol/L	1.976 (0.865, 3.100)^*^	1.912 (0.805, 3.031)^*^	1.905 (0.800, 3.022)^*^

^a^Adjustment was carried out to account for age, sex, residential area, BMI, and SES (income, education, and occupation levels).

^b^Adjustment was further carried out to account for drinking status, smoking status, physical activity level, and the frequencies of fruit and vegetable consumption.

^c^Adjustment was further carried out to account for hypertension.

^*^P < 0.05.

The correlation results between PM_2.5_ exposure and cardiovascular risk factors are shown in Table 3. We found that PM_2.5_ exposure was linked to a higher risk of hypercholesterolemia, hyperbetalipoproteinemia, diabetes and HHcy, and the results of the different models were consistent. After we adjusted for demographic characteristics and behavioral risk factors in the main models, for a 10 μg/m3 increase in PM_2.5_ exposure, the risk of hypercholesterolemia, hyperbetalipoproteinemia, diabetes, and HHcy increased by 7.3% (OR = 1.077, 95% CI = 1.011, 1.146), 19.6% (OR = 1.196, 95% CI = 1.091, 1.312), 4.2% (OR = 1.042, 95% CI = 1.002, 1.084), and 17.1% (OR = 1.171, 95% CI = 1.007, 1.064), respectively. When using the 1-year average exposure as a proxy for measuring long-term exposure, the association between hypoalphalipoproteinemia and PM_2.5_ appeared to be significantly strengthened. The results of sensitivity analyses yielding similar or slightly varying results are presented in the Supplementary Table S3.

**Table 3:** Odds ratios (and 95% CIs) of cardiovascular risk factors associated with a 10 μg/m^3^ increase in long-term exposure to PM_2.5_.

**PM_2.5_, μg/m^3^**	**Model 1^a^**	**Model 2^b^**	**Model 3^c^**
Hypercholesterolemia	1.076 (1.011, 1.145)^*^	1.077 (1.011, 1.146)^*^	1.073 (1.008, 1.143)^*^
Hypertriglyceridemia	0.998 (0.949, 1.048)	0.997 (0.952, 1.044)	0.986 (0.941, 1.033)
Hypoalphalipoproteinemia	1.009 (0.934, 1.090)	1.008 (0.933, 1.090)	1.007 (0.932, 1.089)
Hyperbetalipoproteinemia	1.193 (1.088, 1.308)^*^	1.196 (1.091, 1.312)^*^	1.195 (1.089, 1.311)^*^
Diabetes	1.038 (0.999, 1.080)	1.042 (1.002, 1.084)^*^	1.045 (1.005, 1.086)^*^
Hyperhomocysteinemia	1.171 (1.133, 1.209)^*^	1.171 (1.007, 1.064)^*^	1.167 (1.130, 1.206)^*^

^a^Adjustment was carried out to account for age, sex, area, BMI, and SES (income, education, and occupation levels).

^b^Adjustment was further carried out to account for drinking status, smoking status, physical activity level, and frequencies of fruit and vegetable consumption.

^c^Adjustment was further carried out to account for hypertension.

^*^P < 0.05.

**Figure 1:**
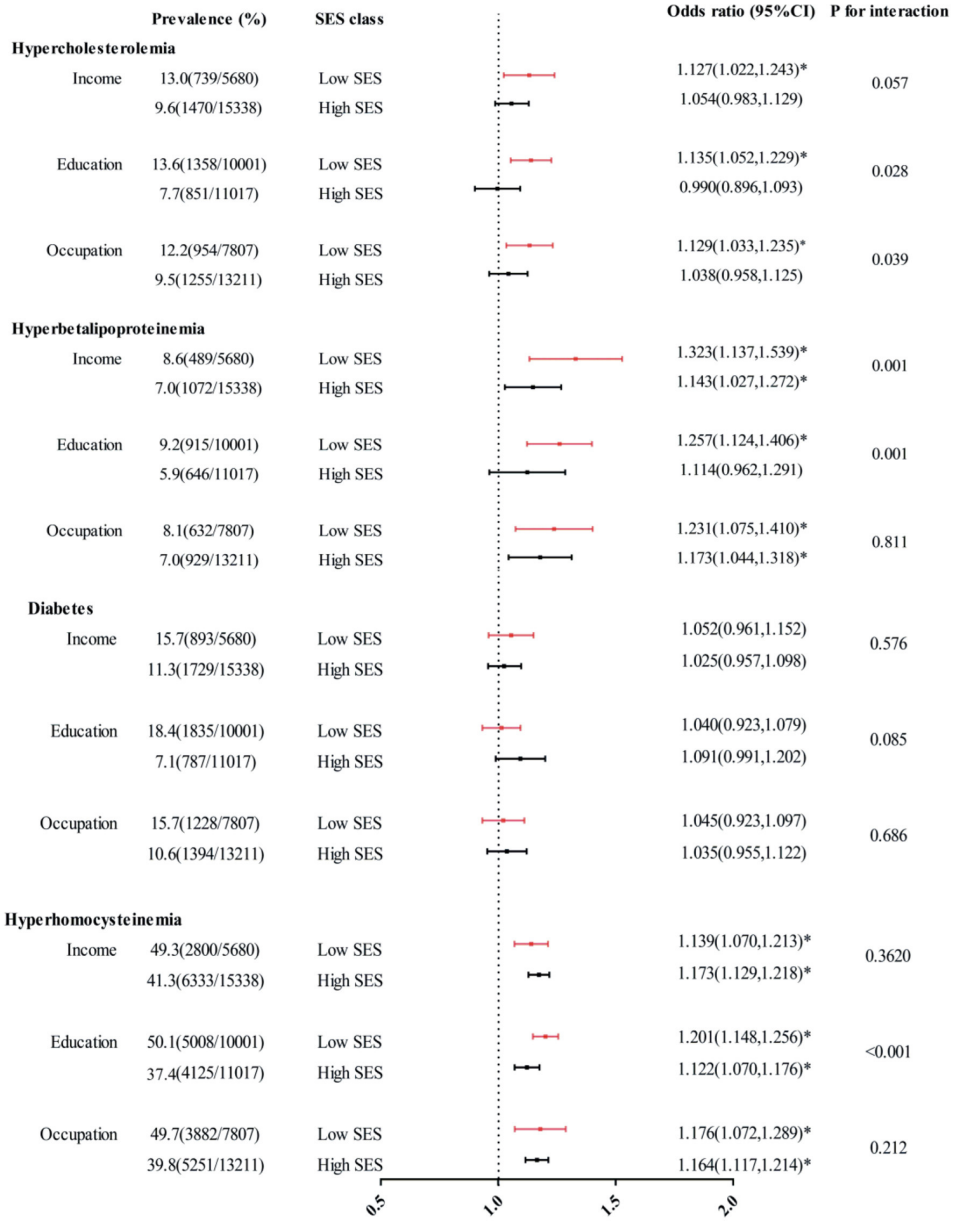
Interaction effects of income, education and occupation levels on associations between each 10 mg/m^3^ increase in PM_2.5_ exposure and hypercholesterolemia, hyperbetalipoproteinemia, diabetes, and HHcy. The covariates included age, sex, location, BMI, smoking status, drinking status, frequencies of fruit and vegetable consumption, and physical activity level. ^*^P < 0.05.

In comparison with those in the high SES group, people in the low SES group were less likely to reach their TC and LDL-C targets (Figure 1). For example, the prevalence of hypercholesterolemia was 12.8%, 13.6% and 12.2% in the low-income, limited education, and mainly manual occupation groups, respectively, while the proportion decreased to 9.7%, 7.7% and 7.5% in the high income, high education, and non-manual occupation groups, respectively. According to our stratified analysis, the effect of PM_2.5_ exposure on health appeared to be greater in patients with a low SES. For hypercholesterolemia, the SES interaction was significant or marginally significant for all three SES indicators (*P* for interaction = 0.058, 0.029, 0.038). For hyperbetalipoproteinemia, the SES interaction was significant for income and education levels (*P* for interaction = 0.009, 0.004) but was not significant for occupation level (*P* for interaction = 0.598). The disparities in statuses did not appear to be significant for the relationship between PM_2.5_ exposure and diabetes risk. Similarly, regarding the relationship between PM_2.5_ exposure and HHcy risk, these disparities manifested only through differences in education level (*P*-value of interaction < 0.001).

**Figure 2:**
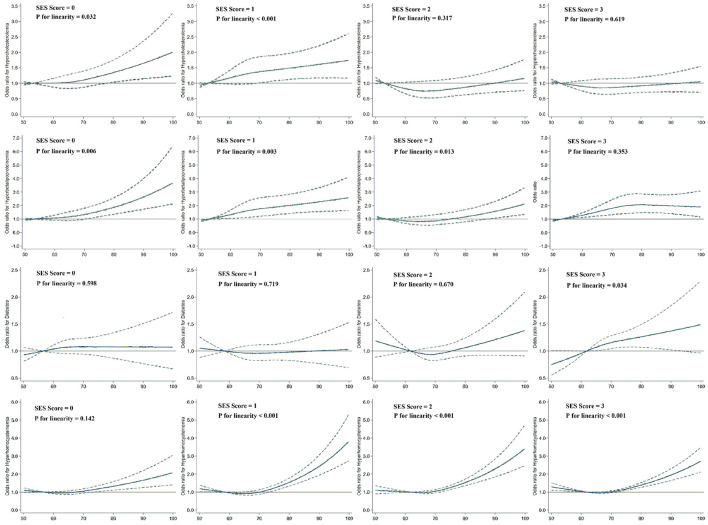
Dose–response relationships of PM_2.5_ exposure with hypercholesterolemia, hyperbetalipoproteinemia, diabetes, and HHcy risk according to SES levels ranging from 1 to 4. Dashed lines represent the 95% CIs of the ORs. Multivariate models were adjusted for age, sex, location, BMI, smoking status, drinking status, physical activity level, and frequencies of fruit and vegetable consumption. The sample sizes for the subgroups were 3,080, 4,511, 5,226, and 8,201.

The relationships between PM_2.5_ exposure and hypercholesterolemia as well as hyperbetalipoproteinemia appeared to increase with decreasing SES (see Supplementary Figure S2). The spline analysis showed a significant linear relationship between PM_2.5_ exposure and dyslipidemia among individuals who exhibited low levels of all three SES indicators (SES score = 0, *P* for linearity = 0.032 for hypercholesterolemia and < 0.001 for hyperbetalipoproteinemia) and among those with a low level for any one of the three SES indicators (SES score = 1, *P* for linearity = 0.006 for hypercholesterolemia and 0.003 for hyperbetalipoproteinemia; Figure 2). No significant linear trend was found between PM_2.5_ exposure and dyslipidemia risk among individuals who exhibited high levels across all three SES indicators (SES score = 3). For diabetes and HHcy risk, the modifying effect of SES was no longer evident. The dose-response curves on PM_2.5_ exposure and diabetes risk exhibited a linear trend exclusively among individuals with an SES level of 3 (*P* for linearity = 0.034). Furthermore, the relationship between PM2.5 exposure and prevalence of HHcy only failed to demonstrate a linear trend among individuals with an SES level of 0 (*P* for linearity = 0.142), whereas it did exhibit such a trend within other SES groups.

## 4 Discussion

Our study substantiates that long-term exposure to PM_2.5_ could lead to elevations in TC, LDL-C, FBG, and Hcy levels. In addition, we observed socioeconomic inequalities in the associations between PM_2.5_ exposure and prevalence of dyslipidemia among adults. Most significantly, our study represents the first comprehensive community-based investigation to assess the modifying effects of SES on the correlation between PM_2.5_ exposure and cardiovascular risk factors. Here, we employed both single-indicator variables, spanning the three aspects of SES, and a composite index for assessment of the effect modification.

Consistent with previous results (44–47), we found that PM_2.5_ was linked to altered TC and LDL-C levels. For example, researchers (45) performed a longitudinal quasiexperiment and found that a reduction in PM_2.5_ corresponded to a significant decrease in TC and LDL-C concentrations, but no significant associations were observed with TG and HDL-C concentrations. Another recent study (46) in China showed that each 10 μg/m^3^ increase in PM_2.5_ was linked to 0.9% (95% CI = 0.6%, 1.2%) and 3.0% (95% CI = 2.6%, 3.5%) increases in TC and LDL-C concentrations, respectively. However, the results of the following studies were less consistent: among conscripted Korean soldiers, long-term exposure to PM_2.5_ was associated with lower HDL-C levels but not with TC, TG or HDL-C levels (15). Among children and adolescents in China, PM_2.5_ concentrations were associated with higher TC concentrations, while no associations were found with other blood lipids (48). This inconsistency could potentially be attributed to the diversity among the research participants (e.g., genetic background and age), study region (e.g., air pollution levels and compositions of PM), and accuracy of the measurements of confounding factors inherent to observational studies (e.g., nutritional status, physical activity level and SES), which, we supposed, were adjusted sufficiently in our current study. In addition, compared with the abovementioned studies, our study has advantages in terms of measuring exposure, which was approximated by calculating a combination of linear concentrations at different locations, accounting for the duration of time spent at home and at work; thus, we believe that the present estimates are more precise.

Specifically, inhalation of particles and gases can induce inflammation and oxidative stress in lung function, which are driven by alveolar macrophages and lung epithelial cells. These effects can be transferred from the lung to the circulatory system, further interfering with lipoprotein metabolism and oxidative stress. In addition, evidence from several previous studies (49, 50) suggests that PM_2.5_ exposure can also cause abnormal DNA methylation by reducing DNA methyltransferase activity. In the present study, the correlations between atmospheric pollutant concentrations and lipid concentrations (especially TC and LDL-C) are consistent with these hypothesized biological mechanisms.

Our study confirmed a substantial association between PM_2.5_ and elevated FBG levels, as well as an increased risk of diabetes outcomes. Several previous epidemiological researches have investigated the impacts of ambient air pollution exposure on the likelihood of developing diabetes. A cross-sectional study (51) conducted in India did not establish a correlation between residential PM_2.5_ exposure and the prediabetes/diabetes incidence in that population. However, studies conducted in East Asia support a relationship between PM_2.5_ exposure and diabetes risk. A study among older adults ≥65 years old in Hong Kong (52) revealed that for every increment of 3.2 μg/m^3^ in ambient PM_2.5_ exposure, there was a corresponding rise of 5% in the OR for diabetes, with a 95% CI ranging from 1.01 to 1.10. Another recent study conducted in Okayama City, Japan (53), revealed that the OR remained significant at 1.10 (1.00–1.20) for each 2.1 μg/m^3^ increment in the interquartile range of the PM_2.5_ concentration. The results of this research underscore the significant impact of PM_2.5_ exposure on diabetes risk, even in areas with relatively low pollution levels.

Despite the lack of a complete understanding of the specific biological mechanisms through which exposure to air pollutants induces diabetes, several theories have been suggested. One such theory postulates that exposure to air pollutants might increase oxidative stress and inflammation within adipose tissue. This, in turn, can trigger endoplasmic reticulum stress, disrupt insulin signaling, and ultimately lead to cell apoptosis. These processes can potentially impact insulin resistance as well as the risk of metabolic disorders (54, 55). PM_2.5_ has also been shown to mediate dysfunction in brown adipose tissue and mitochondria, which are systemic pathologies associated with type 2 diabetes (56). Additionally, air pollutants can cause imbalances in the autonomic nervous system, directly affecting insulin resistance (57). The positive correlation identified in our study between exposure to PM_2.5_ and FBG as well as diabetes, further reinforces these underlying mechanisms and provides compelling proof of the detrimental impacts of exposure to PM_2.5_ on diabetes.

Elevated Hcy levels are acknowledged to be an independent contributory factor for CVD incidence. Despite a growing body of research examining the impact of air pollution on blood Hcy levels, the results remain ambiguous. A recent systematic review (58) synthesized the available evidence supporting a positive correlation between higher concentrations of particulate matter (PM) and elevated Hcy levels. However, owing to the restricted quantity of accessible studies and the significant heterogeneity in terms of study participants, research design, exposure duration, and the composition and sources of particulate matter, we failed to draw a definitive conclusion. In this study, we identified a clear linear correlation between PM_2.5_ exposure and Hcy levels as well as HHcy outcome, providing further demonstration for the relationship between air pollution and health outcomes, especially in areas with high HHcy incidence and concentrated air pollution.

Our results of effect modification by SES are in accordance with the hypothesis that individuals with low SES appear to be disproportionately affected by adverse pollutant-related health effects. This is the first study to assess the role of SES in the correlation between PM_2.5_ exposure and cardiovascular risk factors; thus, it is difficult for us to compare our results. The impact of SES as an effect modifier in the risk of cardiovascular diseases (59, 60), respiratory diseases (25, 26, 61, 62) and all-cause mortality (63, 64) has only recently been investigated. Unlike previous studies that have typically utilized single SES variables without delving into the complex pathways and observations through which SES may impact health and the estimated relationship between exposure to pollutants and health, our study took a comprehensive approach. In addition, the results of previous studies exploring the role of SES as an effect modifier of pollutant-related health effects seem too weak. While some studies have reported that individuals with lower SES experience more pronounced health effects from exposure to pollutants, this relationship has not been consistently observed across all studies. For example, studies in Western Europe (61), Korea (64) and China (63) revealed differential susceptibilities to pollutant-related health effects according to educational and occupational levels. However, none of these differential effects of SES variables had statistically significant interactions, which was probably related to differences in political and cultural factors, direct and indirect measures of SES and lack of power due to limited sample sizes (16). The current study advances previous work by evaluating the multiple facets of SES. Although single SES variables are preferred for statistical analysis, the composite index score (which captures more information) has advantages in stratified analyses and in the communication of results.

In the present study, we found that low SES significantly increased the impact of PM_2.5_ on the risk of hypercholesterolemia and hyperbetalipoproteinemia, while no such association was observed with high SES. The implications of our findings are illustrated in the Graphical Abstract. SES may lie along the causal pathway that links PM_2.5_ exposure to dyslipidemia. Individuals of lower SES not only have fewer resources but also face multiple cooccurring risk factors, including restricted chances of engaging in health-enhancing activities, increased psychosocial stress, and limited access to optimal nutrition. Conversely, deteriorating air quality could impact the desirability of a neighborhood, leading to the migration of higher-SES individuals away from the area while causing lower-SES individuals to move into the area. These findings heighten the susceptibility of individuals with low SES to the detrimental health effects of air pollution exposure. Above all, understanding the directionality of SES and pollutant-related health effects is difficult and important, especially in China, which is a rapidly developing country where different processes may occur in different places.

Based on the findings of this study, the crude prevalence of diabetes among adults aged 18–74 years in Beijing in 2017 was 12.5%. A recent large-scale national survey (65) also revealed that 11.2% of adults aged 18 and over in China suffer from diabetes. The prevalence of diabetes has continued to increase worldwide, and the epidemic trend of diabetes in China is the same as that worldwide, increasing from 9.7% in 2007 and 2010 to 10.4% in 2013 (66–68). According to the results of this study, the overall prevalence of diabetes in China has maintained a continuous growth trend, and there has been no inflection point. The prevalence of diabetes is even greater among people with low income, limited education, or a mainly manual occupation, reaching 15.7%, 18.4%, and 15.7%, respectively. A similar trend was observed for HHcy, and a recent meta-analysis (69) revealed that 37.2% of the Chinese population suffers from HHcy. Our study further indicated that the prevalence of HHcy in Beijing remains at a staggering 43.5%. Moreover, this prevalence increases notably among individuals with low income, education, and occupational statuses, reaching 49.3%, 50.1%, and 49.7%, respectively. However, we failed to verify the modifying effect of SES on the relationship between PM_2.5_ exposure and diabetes or HHcy risk (*P* for interaction > 0.05).

The following are limitations of our study. First, although we evaluated SES as a composite index that included income, education, and occupation levels, it may not fully capture all aspects of SES. This approach, which is common in the literature, omits additional SES aspects (e.g., residence, social support, living environment, community resources, etc.) (16, 18). Second, many adjustments (e.g., physical activity, smoking status and drinking status) were self-reported and only assessed at baseline; thus, evaluation biases were inevitable. Future studies with follow-up investigation data will be necessary. Finally, despite controlling for key covariates in our regression and stratified analysis, residual confounding from unmeasured or unadjusted factors may still be present, potentially overestimating or underestimating the role of SES in the association between PM_2.5_ exposure and the incidence of cardiovascular risk factors.

## 5 Conclusion

In this large-scale community-based study, we observed that PM_2.5_ was associated with unfavorable lipid profiles, elevated FGB and Hcy concentrations, and an increased prevalence of cardiovascular risk factors. Notably, low SES significantly amplified the adverse effect of PM_2.5_ on dyslipidemia. Our findings emphasize the need to improve socioeconomic inequality among adults in Beijing and contribute to the understanding of the urgency of protecting the health of vulnerable groups. In the future, exploring additive interaction models across multiple SES indicators in diverse study areas may be crucial.

## Data availability statement

The raw data supporting the conclusions of this article will be made available by the authors, without undue reservation.

## Ethics statement

The protocol of this work was approved by the Beijing Center for Disease Prevention and Control Ethics Committee [No. 2017D (6)]. The studies were conducted in accordance with the local legislation and institutional requirements. The participants provided their written informed consent to participate in this study.

## Author contributions

JD: Data curation, Formal analysis, Investigation, Methodology, Writing – original draft. BS: Resources, Writing – review & editing. YG: Investigation, Supervision, Writing – review & editing. ZW: Investigation, Supervision, Writing – review & editing. YZ: Data curation, Visualization, Writing – review & editing. HL: Resources, Validation, Writing – review & editing. JL: Resources, Validation, Writing – review & editing. GL: Conceptualization, Funding acquisition, Project administration, Supervision, Writing – review & editing.

## References

[1] Evans MA Sano S Walsh K. Cardiovascular disease, aging, and clonal hematopoiesis. Annu Rev Pathol. (2020) 15:419–38. 10.1146/annurev-pathmechdis-012419-032544 31689371 PMC7104598

[2] Vaduganathan M Mensah GA Turco JV Fuster V Roth GA. The global burden of cardiovascular diseases and risk: a compass for future health. J Am Coll Cardiol. (2022) 80:2361–71. 10.1016/j.jacc.2022.11.005 36368511

[3] Stamler J Daviglus ML Garside DB Dyer AR Greenland P Neaton JD. Relationship of baseline serum cholesterol levels in 3 large cohorts of younger men to long-term coronary, cardiovascular, and all-cause mortality and to longevity. JAMA. (2000) 284:311–8. 10.1001/jama.284.3.311 10891962

[4] Pirillo A Casula M Olmastroni E Norata GD Catapano AL. Global epidemiology of dyslipidaemias. Nat Rev Cardiol. (2021) 18:689–700. 10.1038/s41569-021-00541-4 33833450

[5] Cerin E Chan YK Symmons M Soloveva M Martino E Shaw JE. Associations of the neighbourhood built and natural environment with cardiometabolic health indicators: a cross-sectional analysis of environmental moderators and behavioural mediators. Environ Res. (2024) 240(Pt 2):117524. 10.1016/j.envres.2023.117524 37898226

[6] Guéant JL Guéant-Rodriguez RM Oussalah A Zuily S Rosenberg I. Hyperhomocysteinemia in cardiovascular diseases: revisiting observational studies and clinical trials. Thromb Haemost. (2023) 123:270–82. 10.1055/a-1952-1946 36170884

[7] Baik I. Dietary and modifiable factors contributing to hyper-LDL-cholesterolemia prevalence in nationwide time series data and the implications for primary prevention strategies. Nutr Res Pract. (2020) 14:62–9. 10.4162/nrp.2020.14.1.62 32042375 PMC6997138

[8] Krittanawong C Qadeer YK Hayes RB Wang Z Virani S Thurston GD. PM_2.5_ and cardiovascular health risks. Curr Probl Cardiol. (2023) 48:101670. 10.1016/j.cpcardiol.2023.101670 36828043

[9] de Bont J Jaganathan S Dahlquist M Persson Å Stafoggia M Ljungman P. Ambient air pollution and cardiovascular diseases: an umbrella review of systematic reviews and meta-analyses. J Intern Med. (2022) 291:779–800. 10.1111/joim.13467 35138681 PMC9310863

[10] Valdés S Doulatram-Gamgaram V Maldonado-Araque C García-Escobar E García-Serrano S Oualla-Bachiri W. Association between exposure to air pollution and blood lipids in the general population of Spain. Eur J Clin Invest. (2024) 54:e14101. 10.1111/eci.14101 37795744

[11] Wu G Cai M Wang C Zou H Wang X Hua J. Ambient air pollution and incidence, progression to multimorbidity and death of hypertension, diabetes, and chronic kidney disease: a national prospective cohort. Sci Total Environ. (2023) 881:163406. 10.1016/j.scitotenv.2023.163406 37054795

[12] Wong ND Sattar N. Cardiovascular risk in diabetes mellitus: epidemiology, assessment and prevention. Nat Rev Cardiol. (2023) 20:685–95. 10.1038/s41569-023-00877-z 37193856

[13] Hung HM Chen MF Lee HF Wang HL. Exploration of inflammatory biomarkers and psychological cardiovascular disease risk factors among community dwelling adults: a gender comparison study. Biol Res Nurs. (2024) 26:139–49. 10.1177/10998004231197845 37603875

[14] Gaio V Roquette R Dias CM Nunes B. Ambient air pollution and lipid profile: Systematic review and meta-analysis. Environ Pollut. (2019) 254(Pt B):113036. 10.1016/j.envpol.2019.113036 31465899

[15] Kim KN Ha B Seog W Hwang IU. Long-term exposure to air pollution and the blood lipid levels of healthy young men. Environ Int. (2022) 161:107119. 10.1016/j.envint.2022.107119 35123376

[16] Hajat A MacLehose RF Rosofsky A Walker KD Clougherty JE. Confounding by socioeconomic status in epidemiological studies of air pollution and health: challenges and opportunities. Environ Health Perspect. (2021) 129:65001. 10.1289/EHP7980 34124937 PMC8202292

[17] Mathiarasan S Hüls A. Impact of environmental injustice on children's health-interaction between air pollution and socioeconomic status. Int J Environ Res Public Health. (2021) 18:795. 10.3390/ijerph18020795 33477762 PMC7832299

[18] Schultz WM Kelli HM Lisko JC Varghese T Shen J Sandesara P. Socioeconomic status and cardiovascular outcomes: challenges and interventions. Circulation. (2018) 137:2166–78. 10.1161/CIRCULATIONAHA.117.029652 29760227 PMC5958918

[19] Karney BR. Socioeconomic status and intimate relationships. Annu Rev Psychol. (2021) 4:391–414. 10.1146/annurev-psych-051920-013658 32886585 PMC8179854

[20] Li L Ouyang F He J Qiu D Luo D Xiao S. Associations of socioeconomic status and healthy lifestyle with incidence of dyslipidemia: a prospective chinese governmental employee cohort study. Front Public Health. (2022) 10:878126. 10.3389/fpubh.2022.878126 35757615 PMC9218108

[21] Wang Y Wang Y Xu H Zhao Y Marshall JD. Ambient air pollution and socioeconomic status in China. Environ Health Perspect. (2022) 130:67001. 10.1289/EHP9872 35674427 PMC9175641

[22] Hajat A Hsia C O'Neill MS. Socioeconomic disparities and air pollution exposure: a global review. Curr Environ Health Rep. (2015) 2:440–50. 10.1007/s40572-015-0069-5 26381684 PMC4626327

[23] Bloemsma LD Gehring U Klompmaker JO Hoek G Janssen NAH Lebret E. Green space, air pollution, traffic noise and cardiometabolic health in adolescents: the PIAMA birth cohort. Environ Int. (2019) 131:104991. 10.1016/j.envint.2019.104991 31302482

[24] Cai Y Hansell AL Blangiardo M Burton PR BioSHaRE de Hoogh K. Long-term exposure to road traffic noise, ambient air pollution, and cardiovascular risk factors in the HUNT and lifelines cohorts. Eur Heart J. (2017) 38:2290–6. 10.1093/eurheartj/ehx263 28575405 PMC5837618

[25] O'Lenick CR Winquist A Mulholland JA Friberg MD Chang HH Kramer MR. Assessment of neighbourhood-level socioeconomic status as a modifier of air pollution-asthma associations among children in Atlanta. J Epidemiol Community Health. (2017) 71:129–36. 10.1136/jech-2015-206530 27422981

[26] Munoz-Pizza DM Villada-Canela M Reyna MA Texcalac-Sangrador JL Osornio-Vargas ÁR. Air pollution and children's respiratory health: a scoping review of socioeconomic status as an effect modifier. Int J Public Health. (2020) 65:649–60. 10.1007/s00038-020-01378-3 32405779

[27] Bevan GH Freedman DA Lee EK Rajagopalan S Al-Kindi SG. Association between ambient air pollution and county-level cardiovascular mortality in the United States by social deprivation index. Am Heart J. (2021) 235:125–31. 10.1016/j.ahj.2021.02.005 33592167

[28] Poulsen AH Sørensen M Hvidtfeldt UA Frohn LM Ketzel M Christensen JH. Air pollution and myocardial infarction; effect modification by sociodemographic and environmental factors. A cohort study from Denmark. Environ Res. (2023) 229:115905. 10.1016/j.envres.2023.115905 37086881

[29] Wu Y Yao H. Income inequality, state ownership, and the pattern of economic growth – a tale of the Kuznets curve for China since 1978. Atl Econ J. (2015) 43:165–80. 10.1007/s11293-015-9451-9

[30] Murphy A Palafox B O'Donnell O Stuckler D Perel P AlHabib KF. Inequalities in the use of secondary prevention of cardiovascular disease by socioeconomic status: evidence from the PURE observational study. Lancet Glob Health. (2018) 6:e292–301. 10.1016/S2214-109X(18)30031-7 29433667 PMC5905400

[31] Naylor-Wardle J Rowland B Kunadian V. Socioeconomic status and cardiovascular health in the COVID-19 pandemic. Heart. (2021) 107:358–65. 10.1136/heartjnl-2020-318425 33452116

[32] Du J Shao B Gao Y Wei Z Zhang Y Li H. Associations of long-term exposure to air pollution with blood pressure and homocysteine among adults in Beijing, China: a cross-sectional study. Environ Res. (2021) 197:111202. 10.1016/j.envres.2021.111202 33894236

[33] Zhang W Du J Li H Yang Y Cai C Gao Q. Multiple-element exposure and metabolic syndrome in Chinese adults: a case-control study based on the Beijing population health cohort. Environ Int. (2020) 143:105959. 10.1016/j.envint.2020.105959 32673904

[34] Joint Committee Issued Chinese Guideline for the Management of Dyslipidemia in Adults. 2016 Chinese guideline for the management of dyslipidemia in adults [in Chinese]. Zhonghua Xin Xue Guan Bing Za. (2016) 44:833–53. 10.3760/cma.j.issn.0253-3758.2016.10.005 27903370

[35] Espírito Santo LR Faria TO Silva CSO Xavier LA Reis VC Mota GA. Socioeconomic status and education level are associated with dyslipidemia in adults not taking lipid-lowering medication: a population-based study. Int Health. (2022) 14:346–53. 10.1093/inthealth/ihz089 31693111 PMC10575599

[36] Hämmig O Bauer GF. The social gradient in work and health: a cross-sectional study exploring the relationship between working conditions and health inequalities. BMC Public Health. (2013) 13:1170. 10.1186/1471-2458-13-1170 24330543 PMC4028882

[37] Tanaka H Mackenbach JP Kobayashi Y. Trends and socioeconomic inequalities in self-rated health in Japan, 1986-2016. BMC Public Health. (2021) 21:1811. 10.1186/s12889-021-11708-6 34625032 PMC8501722

[38] Omer W Al-Hadithi T. Developing a socioeconomic index for health research in Iraq. East Mediterr Health J. (2017) 23:670–7. 10.26719/2017.23.10.670 29270967

[39] Lee PH Macfarlane DJ Lam TH Stewart SM. Validity of the International Physical Activity Questionnaire Short Form (IPAQ-SF): a systematic review. Int J Behav Nutr Phys Act. (2011) 8:115. 10.1186/1479-5868-8-115 22018588 PMC3214824

[40] Ranzani OT Bhogadi S Milà C Kulkarni B Balakrishnan K Sambandam S. Association of ambient and household air pollution with lung function in young adults in an peri-urban area of South-India: a cross-sectional study. Environ Int. (2022) 165:107290. 10.1016/j.envint.2022.107290 35594814

[41] Markevych I Zhao T Fuertes E Marcon A Dadvand P Vienneau D. Residential greenspace and lung function decline over 20 years in a prospective cohort: The ECRHS study. Environ Int. (2023) 178:108036. 10.1016/j.envint.2023.108036 37336027

[42] Squillacioti G Bellisario V Ghelli F Marcon A Marchetti P Corsico AG. Air pollution and oxidative stress in adults suffering from airway diseases. Insights from the Gene Environment Interactions in Respiratory Diseases (GEIRD) multi-case control study. Sci Total Environ. (2024) 909:168601. 10.1016/j.scitotenv.2023.168601 37977381

[43] Peng W Liang XD Zhang X Huang XY Lu B Fu Q. Application of physical filter initialization in 4DVar. Monthly Weather Review. (2017) 145. 10.1175/MWR-D-16-0274.135865671

[44] Yang BY Bloom MS Markevych I Qian ZM Vaughn MG Cummings-Vaughn LA. Exposure to ambient air pollution and blood lipids in adults: the 33 Communities Chinese Health Study. Environ Int. (2018) 119:485–92. 10.1016/j.envint.2018.07.016 30048882

[45] Li J Yao Y Xie W Wang B Guan T Han Y. Association of long-term exposure to PM2.5 with blood lipids in the Chinese population: findings from a longitudinal quasi-experiment. Environ Int. (2021) 151:106454. 10.1016/j.envint.2021.106454 33676285

[46] Wang L Chen G Pan Y Xia J Chen L Zhang X. Association of long-term exposure to ambient air pollutants with blood lipids in Chinese adults: the China Multi-Ethnic Cohort study. Environ Res. (2021) 197:111174. 10.1016/j.envres.2021.111174 33894235

[47] Bind MA Peters A Koutrakis P Coull B Vokonas P Schwartz J. Quantile regression analysis of the distributional effects of air pollution on blood pressure, heart rate variability, blood lipids, and biomarkers of inflammation in elderly american men: the Normative Aging Study. Environ Health Perspect. (2016) 124:1189–98. 10.1289/ehp.1510044 26967543 PMC4977045

[48] Gui ZH Yang BY Zou ZY Ma J Jing J Wang HJ. Exposure to ambient air pollution and blood lipids in children and adolescents: a national population-based study in China. Environ Pollut. (2020) 266(Pt 3):115422. 10.1016/j.envpol.2020.115422 32829032

[49] Chen R Meng X Zhao A Wang C Yang C Li H. DNA hypomethylation and its mediation in the effects of fine particulate air pollution on cardiovascular biomarkers: a randomized crossover trial. Environ Int. (2016) 94:614–9. 10.1016/j.envint.2016.06.026 27397927

[50] Bind MA Lepeule J Zanobetti A Gasparrini A Baccarelli A Coull BA. Air pollution and gene-specific methylation in the Normative Aging Study: association, effect modification, and mediation analysis. Epigenetics. (2014) 9:448–58. 10.4161/epi.27584 24385016 PMC4053463

[51] Curto A Ranzani O Milà C Sanchez M Marshall JD Kulkarni B. Lack of association between particulate air pollution and blood glucose levels and diabetic status in peri-urban India. Environ Int. (2019) 131:105033. 10.1016/j.envint.2019.105033 31376594 PMC6718580

[52] Qiu H Schooling CM Sun S Tsang H Yang Y Lee RS. Long-term exposure to fine particulate matter air pollution and type 2 diabetes mellitus in elderly: a cohort study in Hong Kong. Environ Int. (2018) 113:350–6. 10.1016/j.envint.2018.01.008 29357993

[53] Tani Y Kashima S Mitsuhashi T Suzuki E Takao S Yorifuji T. Fine particulate matter and diabetes prevalence in Okayama, Japan. Acta Med Okayama. (2023) 77:607–12. 10.18926/AMO/66152 38145934

[54] Andersen ZJ Raaschou-Nielsen O Ketzel M Jensen SS Hvidberg M Loft S. Diabetes incidence and long-term exposure to air pollution: a cohort study. Diabetes Care. (2012) 35:92–8. 10.2337/dc11-1155 22074722 PMC3241311

[55] Fleisch AF Gold DR Rifas-Shiman SL Koutrakis P Schwartz JD Kloog I. Air pollution exposure and abnormal glucose tolerance during pregnancy: the project Viva cohort. Environ Health Perspect. (2014) 122:378–83. 10.1289/ehp.1307065 24508979 PMC3984217

[56] Purkayastha S Zhang G Cai D. Uncoupling the mechanisms of obesity and hypertension by targeting hypothalamic IKK-β and NF-κB. Nat Med. (2011) 17:883–7. 10.1038/nm.2372 21642978 PMC3134198

[57] Liu C Yang C Zhao Y Ma Z Bi J Liu Y. Associations between long-term exposure to ambient particulate air pollution and type 2 diabetes prevalence, blood glucose and glycosylated hemoglobin levels in China. Environ Int. (2016) 92–93:416–21. 10.1016/j.envint.201603.028 27148900 PMC4902714

[58] Yang BY Shi TX Luo YN Liu XX Zhao T Bloom MS. Ambient air pollution and homocysteine: current epidemiological evidence and a call for further research. Environ Res. (2020) 187:109679. 10.1016/j.envres.2020.109679 32454311

[59] Li R Hou J Tu R Liu X Zuo T Dong X. Associations of mixture of air pollutants with estimated 10-year atherosclerotic cardiovascular disease risk modified by socio-economic status: the Henan Rural Cohort Study. Sci Total Environ. (2021) 793:148542. 10.1016/j.scitotenv.2021.148542 34174609

[60] Chi GC Hajat A Bird CE Cullen MR Griffin BA Miller KA. Individual and neighborhood socioeconomic status and the association between air pollution and cardiovascular disease. Environ Health Perspect. (2016) 124:1840–7. 10.1289/EHP199 27138533 PMC5132637

[61] Keidel D Anto JM Basagaña X Bono R Burte E Carsin AE. The role of socioeconomic status in the association of lung function and air pollution-a pooled analysis of three adult ESCAPE cohorts. Int J Environ Res Public Health. (2019) 16:1901. 10.3390/ijerph16111901 31146441 PMC6603717

[62] Alwahaibi A Zeka A. Respiratory and allergic health effects in a young population in proximity of a major industrial park in Oman. J Epidemiol Community Health. (2016) 70:174–80. 10.1136/jech-2015-205609 26359504 PMC4752616

[63] Xu M Sbihi H Pan X Brauer M. Modifiers of the effect of short-term variation in PM_2.5_ on mortality in Beijing. China Environ Res. (2020) 183:109066. 10.1016/j.envres.2019.109066 32058147

[64] Kim H Byun G Choi Y Kim S Kim SY Lee JT. Effects of long-term exposure to air pollution on all-cause mortality and cause-specific mortality in seven major cities of South Korea: Korean national health and nutritional examination surveys with mortality follow-up. Environ Res. (2021) 192:110290. 10.1016/j.envres.2020.110290 33027629

[65] Li Y Teng D Shi X Qin G Qin Y Quan H. Prevalence of diabetes recorded in mainland China using 2018 diagnostic criteria from the American Diabetes Association: national cross sectional study. BMJ. (2020) 369:m997. 10.1136/bmj.m997 32345662 PMC7186854

[66] Yang W Lu J Weng J Jia W Ji L Xiao J. Prevalence of diabetes among men and women in China. N Engl J Med. (2010) 362:1090–101. 10.1056/NEJMoa0908292 20335585

[67] Wang L Gao P Zhang M Huang Z Zhang D Deng Q. Prevalence and ethnic pattern of diabetes and prediabetes in China in 2013. JAMA. (2017) 317:2515–23. 10.1001/jama.2017.7596 28655017 PMC5815077

[68] Xu Y Wang L He J Bi Y Li M Wang T. Prevalence and control of diabetes in Chinese adults. JAMA. (2013) 310:948–59. 10.1001/jama.2013.168118 24002281

[69] Zeng Y Li FF Yuan SQ Tang HK Zhou JH He QY. Prevalence of hyperhomocysteinemia in China: an updated meta-analysis. Biology. (2021) 10:959. 10.3390/biology10100959 34681058 PMC8533293

