# Supplementary material for: Relationship between exposure to fine particulate matter and cardiovascular risk factors and the modifying effect of socioeconomic status: a cross-sectional study in Beijing, China
Source: Front Public Health. 2024 Jul 19;12:1398396. doi: 10.3389/fpubh.2024.1398396 (PMC11294222; doi:10.3389/fpubh.2024.1398396)
Supplement: Supplementary file 1 [file Data_Sheet_1.docx]

Supplement


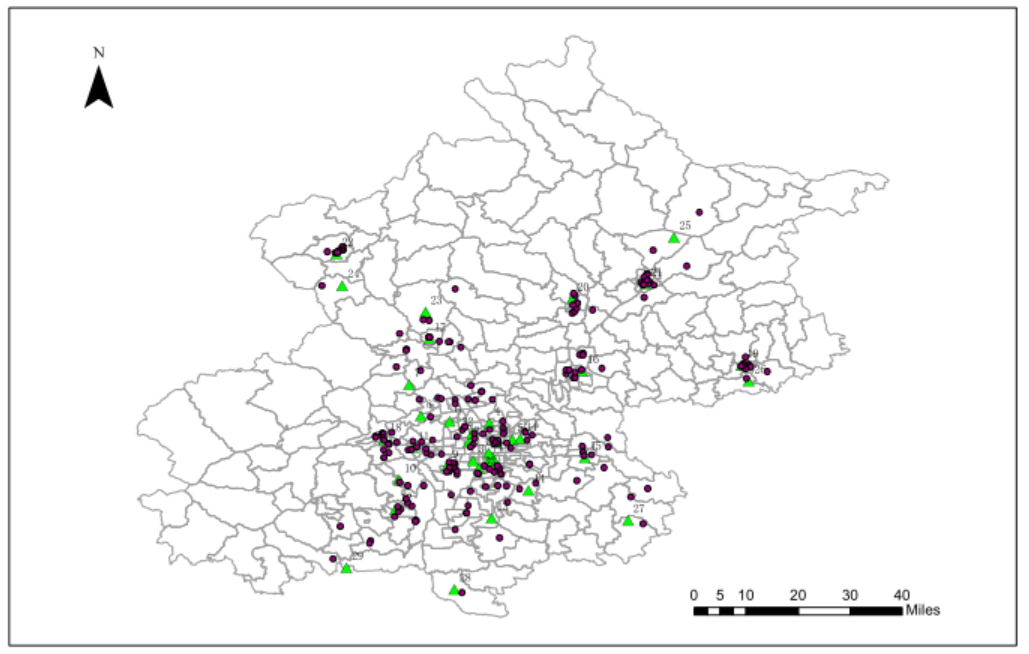


Figure S1 Locations of the study sites and air monitoring stations in Beijing.


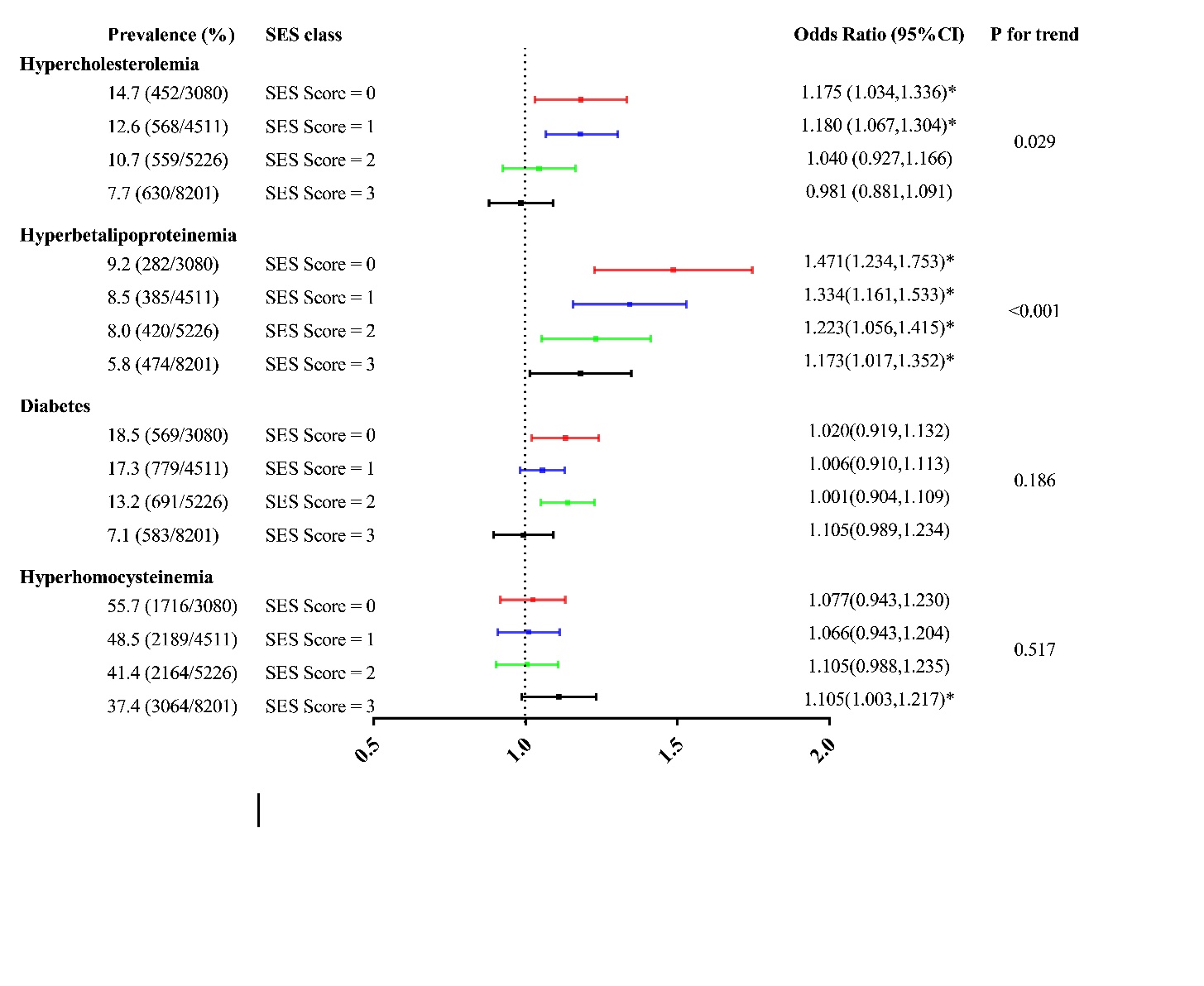


Figure S2. Interaction effects of SES levels on associations between each 10 mg/m^3^ increase in PM_2.5_ exposure and hypercholesterolemia, hyperbetalipoproteinemia, diabetes, and HHcy. The covariates included age, sex, location, BMI, smoking status, drinking status, frequencies of fruit and vegetable consumption, and physical activity level.

**Method S1. Explanation of PM_2.5_ data**

The operation of the monitoring stations strictly followed the methodological standards set by the State Environmental Protection Administration of China (SEPA, 1992).

1. The analysis method

PM_2.5_ was assessed by $\beta$-attenuation. One-hour average mean value is calculated by averaging all the valid one-time values within one hour. At least 75% of the one-time values should be used to calculate the one-hour average mean value.

2) Quality assurance/Quality control

The environmental monitoring centers conducted regularly performance audits and precision checks on the air-monitoring equipment. Quarterly performance audits are conducted to assess data accuracy on PM_2.5_ monitoring systems.

3) Outliers

When the measured concentration is too low (e.g. background value), or a negative value can be obtained because of the zero drift of the monitor. The data was regarded as invalid data.

The data during the period of zero calibration/span calibration becomes out of control until the equipment is recovered was regarded as invalid data.

The data received by the central control station during the period of the loss of power should be regarded as invalid data.

**Method S2. Detailed information on linear mixed regression model and two-level logistic regression models**

We applied linear mixed regression models and two-level logistic regression models

to investigate associations between air pollutant and TC, TG, HDL-C, LDL-C, FBG, and Hcy levels and cardiovascular risk factors. We modeled the outcome variable as a function of *k* covariates (*X_1_*, …, *X_k_*) as follows:

Cardiovascular metabolic indicators: Y_ij_ = $\alpha$_j_+β_1_ X_1ij_ + … + β_k_ X_kij_+ε_ij_ (a)

logit [Probability (Y_ij_)] = $\alpha$^′^_j_+β^′^_1_ X_1ij_ + … + β^′^_k_ X_kij_+ε^′^_ij_ (b)

Where the outcome variable (Y) in equation (b) is cardiovascular risk factors (hypercholesterolemia, hyperbetalipoproteinemia, diabetes, and hyperhomocysteinemia), the subscript j is for communities or villages (j=1,$\cdots$,215), the subscript i is for a participant (i=1,$\cdots{,n}_{j}$), $\alpha$_j_ and $\alpha'$_j_ are the random intercepts at the community level (level 2) assumed to have means of zero and constant variance, β_1_…β_k_ and β^′^_1_…β^′^_k_ are the regression coefficients of the covariates, ε_ij_ and ε^′^_ij_ are random errors at the individual level (level 1) assumed to have means of zero and constant variance.

Table S1. Baseline characteristics of study participants between included and excluded participants

| Characteristics | Total  (N=24990) | Excluded Participants  (N=3972) | Included Participants  (N=21018) | P value |
| --- | --- | --- | --- | --- |
| Age (years), mean (± SD) | 45.8(14.4) | 44.5(15.0) | 46.0(14.3) | <0.001 |
| Male, n (%) | 11669(46.7) | 1820(45.8) | 9849(46.9) | 0.229 |
| Married, n (%) | 21138(84.6) | 3218(81.0) | 17920(85.3) | <0.001 |
| BMI (kg/m2), mean (± SD) | 25.3(7.7) | 25.3(8.5) | 25.3(7.6) | 0.903 |
| Smoking, n (%) |  |  |  | <0.001 |
| Non smoker | 17707(70.8) | 2872(72.3) | 14835(70.6) |  |
| Former smoker | 1344(5.4) | 192(4.8) | 1152(5.5) |  |
| Current smoker | 5939(23.8) | 908(22.9) | 5031(23.9) |  |
| Drinking, n (%) |  |  |  | <0.001 |
| Non drinker | 17607(70.5) | 2905(73.1) | 14702(70.0) |  |
| Nonhabitual drinker | 5073(20.3) | 740(18.6) | 4333(20.6) |  |
| Habitual drinker | 2310(9.2) | 327(8.2) | 1983(9.4) |  |
| Education, n (%) |  |  |  | 0.027 |
| Low | 11570 (46.3) | 1569(39.5) | 10001 (47.6) |  |
| High | 12892 (51.6) | 1875(47.2) | 11017 (52.4) |  |
| Unknown | 528 (2.1) | 528(13.3) | 0 (0.0) |  |
| Income, n (%) |  |  |  | 0.519 |
| Low | 6314 (25.2) | 670(16.9) | 5644 (26.8) |  |
| High | 17256(69.1) | 1882(47.4) | 15374 (73.2) |  |
| Unknown | 1420(5.7) | 1420(35.8) | 0 (0.0) |  |
| Occupation, n (%) |  |  |  | <0.001 |
| Maily manual | 8972(35.9) | 1165(29.3) | 7807 (37.1) |  |
| Non-manual | 14446(57.8) | 1235(31.1) | 13211 (62.9) |  |
| Unknown | 1572(6.3) | 1572(39.6) | 0 (0.0) |  |

Table S2. Baseline characteristics of study participants across different SES

| Characteristics | Total (N=21018) | SES Class=0  (N=3080) | SES Class =1  (N=4511) | SES Class =2  (N=5226) | SES Class=3  (N=8201) |
| --- | --- | --- | --- | --- | --- |
| Age (years), mean (± SD) | 46.0(14.3) | 57.5(10.9) | 51.5(13.7) | 46.5(14.8) | 38.2(10.5) |
| Male, n (%) | 9849(46.9) | 1498(48.6) | 2336(51.8) | 2415(46.2) | 3600(43.9) |
| Married, n (%) | 17920(85.3) | 2776(90.1) | 3957(87.7) | 4413(84.4) | 6774(82.6) |
| BMI (kg/m^2^), mean (± SD) | 25.3(7.6) | 25.8(3.9) | 25.8(8.4) | 25.3(4.4) | 24.8(9.5) |
| Smoking, n (%) |  |  |  |  |  |
| Non smoker | 14835(70.6) | 1877(60.9) | 2832(62.8) | 3768(72.1) | 6358(77.5) |
| Former smoker | 1152(5.5) | 276(9.0) | 307(6.8) | 247(4.7) | 322(3.9) |
| Current smoker | 5031(23.9) | 927(30.1) | 1372(30.4) | 1211(23.2) | 1521(18.6) |
| Drinking, n (%) |  |  |  |  |  |
| Nondrinker | 14702(70) | 2005(65.1) | 3063(67.9) | 3806(72.8) | 5828(71.1) |
| Nonhabitual drinker | 4333(20.6) | 460(14.9) | 830(18.4) | 1012(19.4) | 2031(24.8) |
| Habitual drinker | 1983(9.4) | 615(20.0) | 618(13.7) | 408(7.8) | 342(4.2) |
| Physical activity, n (%) |  |  |  |  |  |
| Low | 5047(24) | 527(17.1) | 1050(23.3) | 1266(24.2) | 2204(26.9) |
| Moderate | 6352(30.2) | 763(24.8) | 1183(26.2) | 1545(29.6) | 2861(34.9) |
| High | 9619(45.8) | 1790(58.1) | 2278(50.5) | 2415(46.2) | 3136(38.2) |
| Low income, n (%) | 5680(27) | 3080(100.0) | 1333(29.6) | 1267(24.2) | 0(0.0) |
| Low education, n (%) | 10001(47.6) | 3080(100.0) | 4187(92.8) | 2734(52.3) | 0(0.0) |
| Mainly manual occupation, n (%) | 7807(37.1) | 3080(100.0) | 3502(77.6) | 1225(23.4) | 0(0.0) |
| Hypertension, n (%) | 7895(37.6) | 1857(60.3) | 2178(48.3) | 1941(37.1) | 1919(23.4) |
| Diabetes, n (%) | 2622(12.5) | 569(18.5) | 779(17.3) | 691(13.2) | 583(7.1) |
| Hypercholesterolemia, n (%) | 2209(10.5) | 452(14.7) | 568(12.6) | 559(10.7) | 630(7.7) |
| Hypertriglyceridemia, n (%) | 4011(19.1) | 637(20.7) | 942(20.9) | 1026(19.6) | 1406(17.1) |
| Hypoalphalipoproteinemia, n (%) | 2908(13.8) | 379(12.3) | 651(14.4) | 716(13.7) | 1162(14.2) |
| Hyperbetalipoproteinemia, n (%) | 1561(7.4) | 282(9.2) | 385(8.5) | 420(8.0) | 474(5.8) |
| PM_2.5_, μg/m^3^ | 67.3(10.9) | 64.1(11.8) | 67.5(10.5) | 68.7(9.8) | 67.6(11.1) |

Data are presented as the mean ± standard deviation for continuous variables, while for categorical variables, they

are expressed as number (percentage). Abbreviations: SD, standard deviation; BMI, body mass index.

Table S3 Sensitivity analyses of odds ratio (and 95% CI) of cardiovascular risk factors associated with a 10 μg/m^3^ increase in long-term exposure to PM_2.5_

| PM_2.5_, μg/m^3^ | Basic Model^a^  (n=21018) | Multiple pollutants^b^  (n=21018) | Non-CVD^c^  (n=19013) | One-year modeld^d^  (n=21018) | WRF model^e^  (n=21018) |
| --- | --- | --- | --- | --- | --- |
| Hypercholesterolemia | 1.077(1.011,1.146) ^*^ | 1.062(0.989,1.140) | 1.075(1.009,1.145) ^*^ | 1.071(1.013,1.132) ^*^ | 1.054(1.001,1.11) ^*^ |
| Hypertriglyceridemia | 0.997(0.952,1.044) | 0.964(0.908,1.022) | 1.000(0.951,1.052) | 0.997(0.954,1.042) | 0.968(0.924,1.015) |
| Hypoalphalipoproteinemia | 1.008(0.933,1.089) | 1.010(0.927,1.101) | 1.032(0.951,1.120) | 1.094(1.026,1.167) ^*^ | 1.026(0.959,1.097) |
| Hyperbetalipoproteinemia | 1.196(1.091,1.312) ^*^ | 1.202(1.087,1.33) ^*^ | 1.199(1.088,1.321) ^*^ | 1.244(1.150,1.345) ^*^ | 1.159(1.069,1.258) ^*^ |
| Diabetes | 1.042(1.002,1.084) ^*^ | 1.048(0.995,1.104) | 1.043(0.999,1.089) | 1.040(1.000,1.080) ^*^ | 1.041(0.992,1.092) |
| HHcy | 1.171(1.133,1.209) ^*^ | 1.29(1.242,1.340) ^*^ | 1.171(1.131,1.212) ^*^ | 1.197(1.162,1.232) ^*^ | 1.116(1.059,1.177) ^*^ |

^a^ The basic models were adjusted for included age, sex, area, BMI, education, income, and occupation, drinking habit, smoking habit, physical activity level.

^b^ NO_2_ was added as another covariate into the regression models in regard to PM_2.5_.

^c^ Analyses were evaluated by excluding participants with CVD (n=19013).

^d^ One-year average exposure was used as a substitution for long-term exposure to PM_2.5_..

^e^ Two year average exposure to PM_2.5_ was generated by WRF model at participants' home addresses over the study period of 2016-2017.

* P < 0.05.
